# Parenteral nutrition: Revisited

**DOI:** 10.4103/0019-5049.63637

**Published:** 2010

**Authors:** Koneru Veera Raghava Chowdary, Pothula Narasimha Reddy

**Affiliations:** Department of Anaesthesiology, Narayana Medical College, Nellore, Andhra Pradesh - 524 002, India

**Keywords:** Parenteral nutrition, critically ill, BEE, nutrients

## Abstract

The prevalence of malnutrition among critically ill patients, especially those with a protracted clinical course, has remained largely unchanged over the last two decades. The metabolic response to stress, injury, surgery, or inflammation cannot be accurately predicted and these metabolic alterations may change during the course of illness. Both underfeeding and overfeeding are common in intensive care units (ICU), resulting in large energy and other nutritional imbalances. Systematic research and clinical trials on various aspects of nutritional support in the ICU are limited and make it challenging to compile evidence-based practice guidelines.

## INTRODUCTION

The malnutrition among the critically ill patients has not altered in recent years,[1,2] inspite of constant metabolic alterations occurring during the course of disease.[3] The observation by Fohn and Denis that protein hydrolysis leads to gut absorption inspired Henriques and Anderson to administer hydrolysed protein in an animal study, in 1913.[4,5] Elman and Weiner reported on the first successful use of Total Parenteral Nutrition (TPN) in humans, in 1939.[6]

Total Parenteral Nutrition became widely accepted after 1967, when Dudrick *et al*. from the University of Pennsylvania demonstrated that normal growth and development could occur solely with the administration of TPN.[7] Seibert was the first to report that fever following TPN was due to pyrogens from the bacterial contamination of TPN fluids.[8]

The 1970s saw wide use of TPN as the predominant route of nutrition. However, enteral nutrition had unique advantages as compared to TPN, which had additional costs in its monitoring and preparation, There was infection-related risk and this involved additional expenditure. All these saw a resurgent advocation of enteral nutrition in 1990.[9]

Parenteral nutrition, which was earlier synonymous with TPN is being reviewed with new interest again, with the concept of partial parenteral nutrition, that is, simultaneous intravenous nutrition with enteral nutrition. This concept of partial Parenteral Nutrition (PN) would include nearly 90% of the surgical inpatients and 100% of the patients being anaesthetised, as they would be receiving intravenous fluids perioperatively. This statement emphasises the need for a sound understanding of parenteral nutrition concepts by the anaesthetist.

## DEFINITION

Parenteral nutrition means feeding someone via their blood stream 'intravenously', TPN means feeding a patient solely via the intravenous route.[10]

## INDICATIONS

- When patient gastrointestinal tract is paralysed and nonfunctional, as in the case of small bowel obstruction
- When >7 days of nothing-by-mouth (NPO) status is anticipated, as in the case of inflammatory bowel disease, patients with an acute exacerbation, critically ill patients and so on
- When the baby's gut is too immature or has congenital malformations
- When the patient is suffering from chronic diarrhoea and vomiting or is extremely undernourished and needs to have surgery, chemotherapy and so on
- When patients with bowel anastomoses develop anastomotic leaks in the early postoperative period[11]

## PRACTICAL CONSIDERATIONS IN IV SITE SELECTION

Central veins can handle higher macronutrient concentrations compared to peripheral vessels, without the risk of thrombophlebitis or vessel damage.[12] True peripheral veins cannot tolerate concentrations of more than 900 mosm/L; concentrations of Calcium ≤ 5 mEq/L and Potassium ≤ 40 mEq/L are recommended. Ideally the peripheral veins are suitable for administration of isotonic fat emulsions and hypocaloric dextrose solutions (i.e., < 10% dextrose), hence, they are limited to preventing starvation adaptation and minimizing nitrogen loss.[13]

The Subclavian Vein is the most suitable vein for parenteral nutrition as this is comfortable to the patient and carries less risk of dislodgement compared to the IJV and has less thrombophlebitis risk compared to the Femoral vein. Exclusively dedicated lumen should be available for PN; a central venous line should be placed with strict aseptic precautions after cleaning the skin with chlorhexidine and dressed with sterile dressing every 48 hours.[14] The European Society for Clinical Nutrition and Metabolism (ESPEN) has issued specific guidelines on the use of Central Venous Catheters with regard to access, care, diagnosis and therapy of complications.[15]

## ENERGY REQUIREMENTS AND RECOMMENDATIONS

Indirect calorimetry is considered as the gold standard for the measurement of metabolic rate and substrate utilisation, but the Harris-Benedict equation is a more practical means of estimating the Basal Energy Expenditure (BEE) in adults.[16]

Men : BEE = 66 + 13.8 (wt in Kg) + 5 (Ht in cm) – 6.8 (age)

Women: BEE = 655 + 9.6 (wt in Kg) + 1.8 (Ht in cm) – 4.7 (age)

Ideal body weight (wt) may be calculated using the Hamwi method

Men : 106 lb (for first 5 ft) + 6 lb (for each additional inch past 5 ft)

Women : 100 lb (for first 5 ft) + 5 lb (for each additional inch past 5 ft)

A much easier method is computation of the IBW by Broca's index

Men : Wt in Kg = (Height in cm) – 100

Women: Wt in Kg = (Height in cm) – 105

Stress factors have to be added to this BEE[17‐23] [Table 1]

**Table 1:** Energy requirement recommendations in various clinical situations

Stress factors	% BEE
Elective surgery	110
Medical illness of non-critical nature	120
Trauma	135 – 150
Burns	150 – 160
Sepsis	160 – 180

However, the caloric requirements should be individualised with respect to the degree of stress, organ failure and percentage of ideal body weight. The calories should be provided in the form of carbohydrates, proteins and fats, in the right mixture, so as to achieve a respiratory quotient (RQ) of around 0.8; a lower RQ (< 0.7) indicates fat oxidation and a higher RQ (> 1) indicates Lipogenesis. A reasonable and well-accepted recommendation is to initiate it with 25 cal/Kg/day and 1.25 – 2 g protein/Kg/day. This should be augmented according to the stress levels of the patients.[24‐26]

## FLUID REQUIREMENT

Fluid management in PN depends on the hydration status of the patient and the clinical conditions, such as, renal failure, congestive heart failure and so on. In general the amount of fluid equivalent in millilitres per calculated patients BEE calories constitutes adequate hydration.

Generally TPN orders should be reviewed twice daily on a 12-hour basis, so that changes in electrolytes or acid-base balance can be addressed appropriately without wastage of costly TPN solutions. The total amount of fluid taken is restricted by making the nutritional fluid more hypertonic in patients with renal failure, patients with CHF and so on.

## PROTEIN REQUIREMENTS

The provision of adequate protein as an energy source is necessary for the proper utilisation of amino acids. Healthy adults require 0.8 – 1.0 gm of protein per kilogram per day.[27] Parenteral proteins were earlier provided as casein solutions, which had higher microbicidal growth rates due to contamination, but now they are provided in the form of crystalline amino acids, which have better nitrogen balance and do not promote microbial growth.[28] The amino acid profile is based on the World Health Organisation (WHO) recommendations for adequate essential amino acid proportions.[29]

Critically ill patients, without any hepatic or renal dysfunction, would need about 1.5 gm of protein per kg per day,[30] while patients with chronic renal failure should be given 0.6 – 0.8 gm/kg/day and patients with acute hepatic encephalopathy should have a temporary restriction of protein to 0.8 gm/kg/day.[31‐33] Patients on haemodialysis or peritonealdialysis would require 1.2 – 1.3 gm/kg/day.[34] Patients who receive renal replacement therapy (CRRT, CVVHD, CVVHDF) have daily protein requirements of up to 2.5 gm/kg/day in order to meet the nitrogen balance due to the hypercatabolic nature of acute renal failure and protein losses during filtration.[35]

In case of chronic hepatic encephalopathy, unresponsive to protein restriction and pharmacotherapy, it is advised to include branched chain amino acids and exclude aromatic amino acids, as these patients have an impaired ability to metabolise aromatic amino acids and have a depressed level of branched chain amino acids.[36,37] In sepsis and injury, exogenous branched chain amino acids may improve protein synthesis as they are used preferentially by skeletal muscles when the plasma levels are depressed.[38,39] Glutamine is a conditional amino acid, present during the hypercatabolic state, but absent in the commercially prepared parenteral solutions, as it degrades to toxic pyroglutamic acid, but success has been achieved in administering it in the form dipeptides mixed with parenteral nutrition formulae.[40,41] Several recent studies regarding parenteral supplementation of L-Glutamine and L-alanyl-L-Glutamine in a critical illness have shown improved nitrogen balance, decreased length of stay, decreased infection and reduced mortality, but clear cut data are yet to emerge.[42‐45]

## CARBOHYDRATE REQUIREMENTS

Glycogen stores in the liver and skeletal muscles are depleted within 24 hours; the body converts skeletal muscle protein, mainly glutamine and alanine to glucose via gluconeogenesis to meet the basal metabolic demand. Renal medulla, white and red blood cells, seminiferous tubules and the brain require a continuous supply of glucose (100 – 150 gm/day) as they cannot utilise any other substrate for energy; but recently a consensus is lacking, as a few authors feel the brain can utilise ketone bodies as a substrate for about 70% of the metabolic energy requirements, in extreme situations.[46] The maximum glucose utilisation rate in critical illness is 5 – 7 mg/kg/min, and providing carbohydrates in excess can lead to hyperglycemia, hypertriglyceridemia and an increased RQ of > 1.0; diabetic patients are at a greater risk for the above-mentioned complications.[47,48]

Dextrose monohydrate, in concentrations from 2.5 to 70%, is the most common form in which carbohydrate is administered parenterally. One gram of Dextrose provides 3.4 calories. Most TPN regimens utilise ≤ 25% dextrose, while the PPN utilise ≤ 10% dextrose solutions for safe osmolarity infusion. Fructose, sorbitol, xylitol and glycerol as carbohydrate sources for parenteral nutrition have been and are being studied, but none of them have been seen to have any decisive advantage over dextrose and do not have the US Food and Drug Administration (USFDA) approval for use.[49‐51]

## FAT REQUIREMENTS

Lipids in parenteral nutrition are used to provide calories and prevent essential fatty acid deficiency (EFAD), which may develop within three weeks of fat-free parenteral nutrition. Soybean / safflower oil, egg yolk phospholipids in 10, 20 and 30% concentrations are the common sources for lipids in TPN. Patients can receive 25 to 30% total calories as lipids; it is recommended to limit lipids to 0.1 gm/kg/hour.[52] Patients receiving parenteral intralipids with elevated triglycerides (> 400 mg/dL) must have intralipids checked five hours after the lipid infusion is stopped. If levels continue to be elevated, approximately 500 mL of 10% intralipids or 250 mL of 20% intralipids must be provided over 8 to 10 hours, thrice a week.[53] Five hundred milliliters of 20% intralipids may also be given once per week. Propofol and parenteral intralipids must be used with caution because they are mainly composed of soybean oil or omega-six fatty acids, which have been seen to be immunosuppressive.

Long chain triglycerides (LCT) were the main source of lipids used in TPN earlier, but as subsequent studies have suggested that the LCTs impair the immune system, specifically the reticuloendothelial system (RES), structured lipids, which are a combination of long and medium chain fatty acids have been found to improve liver function tests and maintain the low density lipoprotein : high density lipoprotein ratio (LDL:HDL). Additional alternatives to these structured lipids are Omega-3 fatty acids and short chain fatty acids.[54‐57] Providing 2 – 4% of the total calories. As linoleic acid can prevent the occurrence of EFAD, the use of topical vegetable oils to prevent EFAD is controversial.[58,59] The Centre for Disease Control recommends that intralipid infusions be given within 12 hours once started, to avoid gram-negative sepsis.[60]

## MICRONUTRIENT REQUIREMENTS

Electrolytes, trace elements and vitamins come under micronutrients. Sodium 100 – 150 mEq, Potassium 50 – 100 mEq, Magnesium 8 – 24 mEq, Calcium 10 – 20 mEq and Phosphorous 15 – 30 mEq are recommended per litre of parenteral infusion solution. A total of less than 40 mEq is recommended for calcium and phosphorous to prevent precipitation. Typically, calcium is provided as a gluconate and magnesium is provided as a sulphate, due to improved solubility and compatibility.[61] Renal failure, cardiac problems, intestinal losses, hydration status of the patient along with clinical judgement should be factors in considering altering these normal recommendations for electrolytes [Table 2].

**Table 2:** Recommendations for electrolytes in parenteral solutions

Electrolyte	Recommended daily intake	Effects of serum deficiency	Effects of serum excess
Sodium	100 – 150 mEq	Generalised oedema, confusion, hypotension, irritability, lethargy, seizures	Decreased skin turgor, mild irritability, complaints of thirst, elevated blood urea nitrogen and hematocrit
Potassium	60 – 120 mEq	ECG changes as T wave flattening, prolonged PR interval, prominent U waves, ileus, cardiac dysrhythmias, muscle weakness, negative nitrogen balance	ECG changes as peaked T waves, widened QRS, loss of P wave, loss of R wave amplitude, ventricular fibrillation, asystole, muscle weakness
Calcium	10 – 15 mEq	Parasthesias, irritability, tetany, ventricular arrhythmias	Confusion, dehydration, muscle weakness, nausea, vomiting, coma
Phosphorus	450 – 900 mEq	Muscle weakness, red blood cell rigidity with oxygen-haemoglobin curve shift to left	Parasthesias, flaccid paralysis, mental confusion, hypertension, soft tissue calcification
Magnesium	120 – 240 mEq	Neurological irritability (seizures), neuromuscular irritability (tetany), cardiac dysrhythmias	Respiratory paralysis, hypotension, premature ventricular contractions, liver dysfunction.
Chloride	100 – 150 mEq	Nonspecific symptoms such as lethargy, confusion etc., seen in contraction alkalosis	Non-gap metabolic acidosis
Bicarbonate	As needed to maintain acid base balance	Metabolic acidosis*	Metabolic alkalosis

* Not added to parenteral nutrition solutions as it precipitates with calcium, magnesium and changes the pH of the solution

Copper, zinc, selenium and chromium are the common trace elements that are supplemented in PN. Many of these trace elements are monitored monthly in case of patients on prolonged PN, and subsequent action is taken in the forthcoming month. Manganese and copper may be withheld in patients with hepatic dysfunction, while Selenium and chromium intake is restricted in cases of renal failure.[62‐67] Iron is incompatible with lipid containing formulations of PN and is usually administered as iron dextran in solution containing dextrose.[68‐69] Iodine is often omitted from PN, given that an adequate amount of iodine is absorbed into the skin, due to use of iodine containing disinfectants / detergents used during hospital stay. Molybdenum supplementation is required in neonates / infants on prolonged PN [Table 3].

**Table 3:** Recommendations for trace elements in parenteral solutions

Trace element	Recommended daily intake	Effects of deficiency
Zinc	2 – 4 mg/L (12 – 18 mg/L in small bowel / rectal losses)	Dermatitis, alopecia, impaired wound healing, impaired immune function, gonadal atrophy
Copper	300 – 450 µg	Anaemia, demineralization of bone
Iron	0.5 – 1.5 mg	Anaemia
Chromium	10 – 15 µg	Glucose intolerance, peripheral neuropathy, hyperlipidemia
Manganese	2 – 5 mg	Bleeding disorders, impaired wound healing
Selenium	50 – 100 µg	Cardiomyopathy, myositis, arthritis, hair and nail changes
Molybdenum	10 – 50 µg	Amino acid intolerance

Multivitamin preparations that are commercially available can be added to PN solutions. Many of these lack vitamin K, which needs to be added separately into the PN bag once a week. Thiamine is excessively lost in patients on dialysis and merits individual supplementation in such patients. It is common for intensivists to err on the side of over provision of large amounts of vitamin C, thiamine and perhaps zinc; considering their role in wound healing and improvement in the general condition; but this at times may be deleterious, as excessive amounts of these, especially vitamin C, may lead to increased oxidative stress[70] [Table 4].

**Table 4:** Recommendations for vitamins in parenteral solutions

Vitamin	Recommended daily intake	Effects of deficiency
A	4500 IU	Infections / sepsis
Thiamine (B_1_)	5 mg	Wernicke's encephalopathy, Korsakoff's psychosis, break in Kreb cycle
Pyridoxine (B_6_)	6 mg	Neuropathy, dermatitis, irritability
B_12_	3 µg	Megaloblastic anaemia, glossitis
C	50 mg	Scurvy
D	450 IU	Rickets
E	15 IU	Increased oxidants, dermatitis
K	5 – 10 mg	Decreased levels of coagulation factors II, VII, IX, X
Folic acid	400 µg	Neuropathy, glossitis
Niacin	15 mg	Delerium, confusion, dermatitis, pellegra, stomatitis, diarrhoea
Riboflavin	1.8 mg	Glossitis, cheliosis, pruritis, anogenital inflammation
Pantothinic acid	15 mg	Listlessness, fatigue, irritability, malaise, sleep disturbances, increased insulin senstivity
Biotin	60 µg	Anorexia, pallor, glossitis, seborrheic dermatitis, elevated bile pigments

Table summarizes ESPEN Guidelines for Parenteral Nutrition: Intensive care[71] [Table 5]

**Table 5:** ESPEN recommendations for parenteral nutrition: A summary

Subject	Recommendations
Indications	Patients should be fed because starvation or underfeeding in ICU patients is associated with increased morbidity and mortalitys
	All patients who are not expected to be on normal nutrition within three days should receive PN within 24 to 48 hours if EN is contraindicated or if they cannot tolerate EN
Requirements	ICU patients receiving PN should receive a complete formulation to cover their needs fully
	During acute illness, the aim should be to provide energy as close as possible to the measured energy expenditure, in order to decrease negative energy balance
	In the absence of indirect calorimetry, ICU patients should receive 25 kcal/kg/day increasing to target over the next two to three days
Supplementary PN with EN	All patients receiving less than their targeted enteral feeding after two days should be considered for supplementary PN
Carbohydrates	The minimal amount of carbohydrate required is about 2 g/kg of glucose per day
	Hyperglycemia (glucose > 10 mmol/L) contributes to death in the critically ill patients and should also be avoided to prevent infectious complications
	Reductions and increases in mortality rates have been reported in ICU patients when blood glucose is maintained between 4.5 and 6.1 mmol/L. No unequivocal recommendation on this is therefore possible at present
	There is a higher incidence of severe hypoglycemia in patients treated with tighter limits
Lipids	Lipids should be an integral part of PN for energy and to ensure essential fatty acid provision in long-term ICU patients
	Intravenous lipid emulsions (LCT, MCT or mixed emulsions) can be administered safely at a rate of 0.7 g/kg up to 1.5 g/kg over 12 to 24 hours
	The tolerance of mixed LCT / MCT lipid emulsions in standard use is sufficiently documented. Several studies have shown specific clinical advantages over soybean LCT alone, but require confirmation by prospective controlled studies
	Olive oil-based parenteral nutrition is well-tolerated in critically ill patients.
	Addition of EPA and DHA to lipid emulsions has demonstrable effects on cell membranes and inflammatory processes.
	Fish oil-enriched lipid emulsions probably decrease the length of stay in critically ill patients
Amino acids	When PN is indicated, a balanced amino acid mixture should be infused at approximately 1.3 – 1.5 g/kg ideal body weight / day in conjunction with an adequate energy supply
	When PN is indicated in ICU patients the amino acid solution should contain 0.2 – 0.4 g/kg/day of l-glutamine (e.g., 0.3 – 0.6 g/kg/day alanyl-glutamine dipeptide)
Micronutrients	All PN prescriptions should include a daily dose of multivitamins and trace elements.
Route	A central venous access device is often required to administer the high osmolarity PN mixture designed to completely cover the nutritional needs
	Peripheral venous access devices may be considered as low osmolarity (< 850 mOsmol/L) mixtures, designed to cover a proportion of the nutritional needs and to mitigate negative energy balance
	If peripherally administered PN does not allow full provision of the patient's needs then PN should be centrally administered
Mode	PN admixtures should be administered as a complete all-in-one bag

## INITIATION, MAINTENANCE AND MONITORING OF PARENTERAL NUTRITION

The timing of TPN is a vexing question, it would be prudent to start it as soon as one appreciates that the patient is in requirement of TPN, that is, nutritionally compromised. Strict aseptic precautions should be followed during introduction of the central line; the external dressing should be changed every 48 hours using sterile precautions. The external tubing should be changed every 24 hours starting with the first feed of the day. The lumen being used for TPN should be exclusively reserved for it and no drugs / infusions (except insulin infusion) should be allowed in that lumen.

An interdisciplinary nutrition team, comprising of the treating physician, intensivist, nutritional therapist and critical care nurse should monitor the patient's nutritional status regularly on a day-to-day basis. Progress should be documented on a flow chart in terms of bodyweight, blood counts, serum electrolytes and BUN levels, every 24 hours. Blood sugar levels must be monitored hourly till they are stable, and later six hourly / sos as and when needed once the patient and his insulin therapy have attained equilibrium.[72‐74] The most accurate reflection of a critically ill patient's current nutritional status in respect to protein nutrition is made by measuring the pre-albumin levels.[75‐79] Blood lipid levels may be monitored twice weekly. Liver function tests must be monitored weekly. Patients on long-term TPN need monthly monitoring of vitamin, mineral and trace element status. Monitoring should be highly individualised to the existing needs and co-morbidities of the patient.

## HOME PARENTERAL NUTRITION

Home parenteral nutrition (HPN) was introduced as a treatment modality in the early 1970s, primarily for the treatment of chronic intestinal failure, in patients with benign disease. The relatively low morbidity and mortality associated with HPN has encouraged its widespread use in western countries. Thus, there is a huge clinical experience, but there are still few controlled clinical studies on the treatment effects and management of complications.[79]

## COMPLICATIONS OF TPN

Subtle deterioration of the overall clinical well-being of the patient may be the first clue that a TPN-related complication has occurred. The catheter insertion site infection should be ruled out as the first possibility. In case of any tenderness, redness, drainage, warmth or other inflammatory signs at the site of insertion, a fresh catheter should be re-sited at a different site and the tip of the present catheter along with a wound swab should be sent for culture and sensitivity tests. Differentiation between the systemic inflammatory response syndrome and the actual infection remains a difficult task, although preliminary evidence suggests that new markers such as procalcitonin may be valuable in some circumstances.[80]

Re-feeding syndrome characterised by hypophospatemia, hypomagnesemia, hypokalaemia and hyperinsulinemia may be observed in patients kept NPO for greater than 7 – 10 days, chronic alcoholics and those with severe systemic derangements on initiation of TPN.[81,82] Correction of electrolyte abnormalities, administration of thiamine in alcoholics and a regular check of electrolyte levels may help to prevent this re-feeding syndrome.

Short-term potential adverse effects of PN include: infection, hyperglycemia, hepatic steatosis, essential fatty acid deficiency, electrolyte abnormalities, acid-base disturbances, hypertriglyceridemia, bacterial translocation and compromise of gut integrity. The symptoms of essential fatty acid deficiency include dermatitis, alopecia, poor wound healing, increased platelet aggregation, increased capillary fragility and hepatic dysfunction.[83] Long-term adverse effects including all the above-mentioned short-term complications along with vitamin / mineral deficiency / toxicity, aluminium toxicity in infants / patients, with impaired renal system are well documented.

## FUTURE PROSPECTS AND RESEARCH IN TPN

Standard anthropometric measures may not be accurate in critically ill patients and the existing measurement techniques, such as the DEXA scan (Dual Energy X-ray Absorptiometry), are not cost effective, hence, future research in the validation of simple, noninvasive bedside body composition measurement techniques is required.[3]

Martin *et al*. have demonstrated that Glucagon-like-peptide-2 (GLP-2) alone in parenteral nutrition, without enteral feeding, stimulated indices of intestinal adaptation in an animal study, human trials are awaited.[84] Strong, evidence-based support with regard to immunonutrition, with dipeptide glutamine and alanyl-glutamine, is emerging, and similarly the role of a combination of omega-3-fatty acids and 5-flurouracil combination in patients with cancer are being investigated.[85]

Hence, days of rationalising rather than ritualising parenteral nutrition are seen at the horizon.

## References

[1] Hulst J, Joosten K, Zimmermann L, Hop W, van Buuren S, Büller H (2004). Malnutrition in critically ill children: From admission to 6 months after discharge. Clin Nutr.

[2] Pollack MM, Wiley JS, Kanter R, Holbrook PR (1982). Malnutrition in critically ill infants and children. JPEN J Parenter Enteral Nutr.

[3] Mehta NM, Compher C (2009). A.S.P.E.N Board of DirectorsA. .S.P.E.N. Clinical guidelines: Nutrition support of the critically ill child JPEN J Parenter Enteral Nutr.

[4] Henriques V, Andersen AC (1913). Uber parenterale Ernahrung durch intravenose injection. Zeit Physiol Chem.

[5] Fohn O, Denis W (1913). Protein metabolism from the standpoint of blood and tissue analysis: Absorption from large intestine. J Biol Chem.

[6] Elman R, Weiner DO (1939). Intravenous alimentation with special reference to protein (amino acid) metabolism. J Am Med Assoc.

[7] Dudrick Sj, Wilmore DW, Vars HM (1967). Long-term total parenteral nutrition with growth in puppies and positive nitrogen balance in patients. Surg Forum.

[8] Seibert FF (1924). Fever producing substances found in some distilled water. Am J Physiol.

[9] Braunschweig, Carol L (2001). Enteral compared with parenteral nutrition: a meta-analysis. Am J Clin Nutr.

[10] http://www.pharmainfo.net/reviews/total-parenteral-nutrition-review.

[11] Mirtallo F, Gottschlich M (2001). Introduction to parenteral nutrition. The Science and Practice of Nutrition Support.

[12] Dickerson RN, Brown RO, White KG, Rombeau JL, Caldwell MD (1993). Parenteral Nutrition solutions. Parenteral Nutrition.

[13] Isaacs JW, Millikan WJ, Stackhouse J, Hersh T, Rudman D (1977). Parenteral nutrition of adults with a 900 milliosmolar solution via peripheral veins. Am J Clin Nutr.

[14] Conly JM, Grieves K, Peters B (1989). A prospective, randomized study comparing transparent and dry gauze dressings for central venous catheters. J Infect Dis.

[15] Pittiruti M, Hamilton H, Biffi R, MacFie J, Pertkiewicz  M (2009). ESPEN. ESPEN Guidelines on Parenteral Nutrition: Central Venous Catheters (access, care, diagnosis and therapy of complications). Clin Nutr.

[16] Harris JA, Benedict FG (1919). Biometric Studies of Basal Metabolism in Man Publication No 270.

[17] Long CL, Schaffel N, Geiger JW, Schiller WR, Blakemore WS (1979). Metabolic response to injury and illness: Estimation of energy and protein needs from indirect calorimetry and nitrogen balance. JPEN J Parenter Enteral Nutr.

[18] Frankenfield DC, Wiles CE 3rd, Bagley S, Siegel JH (1994). Relationships between resting and total energy expenditure in injured and septic patients. Crit Care Med.

[19] Weissman C, Kemper M, Damask MC, Askanazi J, Hyman AI, Kinney JM (1984). Effect of routine intensive care interactions on metabolic rate. Chest.

[20] Mann S, Westenskow DR, Houtchens BA (1985). Measured and predicted caloric expenditure in the acutely ill. Crit Care Med.

[21] Kreymann G, Grosser S, Buggisch P, Gottschall C, Matthaei S, Greten H (1993). Oxygen consumption and resting metabolic rate in sepsis, sepsis syndrome, and septic shock. Crit Care Med.

[22] Hunter DC, Jaksic T, Lewis D, Benotti PN, Blackburn GL, Bistrian BR (1998). Resting energy expenditure in the critically ill: estimations versus measurement. Br J Surg.

[23] Boulanger BR, Nayman R, McLean RF, Phillips E, Rizoli SB (1994). What are the clinical determinants of early energy expenditure in critically injured adults?. J Trauma.

[24] (1998). National Advisory Group on Standards and Practice Guidelines for Parenteral Nutrition Special Report: Safe practices for parenteral nutrition formulations. J Parenter Enter Nutr.

[25] Jacobs DG, Jacobs DO, Kudsk KA, Moore FA, Oswanski MF, Poole GV (2004). Practice management guidelines for nutritional support of the trauma patient. J Trauma.

[26] McCowen KC, Friel C, Sternberg J, Chan S, Forse RA, Burke PA (2000). Hypocaloric total parenteral nutrition: Effectiveness in prevention of hyperglycemia and infectious complications: A randomized clinical trial. Crit Care Med.

[27] (1989). National Research Council Recommended daily allowances.

[28] Goldmann DA, Martin WT, Worthington JW (1973). Growth of bacteria and fungi in total parenteral nutrition solutions. Am J Surg.

[29] (1985). WHO (World Health Organisation): Energy and protein requirements: Report of a joint FAO/WHO/UNU expert consultation. Technical Report Series 724.

[30] Shaw JH, Wildbore M, Wolfe RR (1987). Whole body protein kinetics in severely septic patients: The response to glucose infusion and total parenteral nutrition. Ann Surg.

[31] (2000). Clinical practice guidelines for nutrition in chronic renal failure K/DOQI, National Kidney Foundation. Am J Kidney Dis.

[32] Nompleggi DJ, Bonkovsky HL (1994). Nutritional supplementation in chronic liver disease: An analytical review. Hepatology.

[33] Mullen KD, Weber FL (1991). Role of nutrition in hepatic encephalopathy. Semin Liver Dis.

[34] Ikizler TA, Flakoll PJ, Parker RA, Hakim RM (1994). Amino acid and albumin losses during hemodialysis. Kidney Int.

[35] Scheinkestel CD, Kar L, Marshall K, Bailey M, Davies A, Nyulasi I (2003). Prospective randomized trial to assess caloric and protein needs of critically Ill, anuric, ventilated patients requiring continuous renal replacement therapy. Nutrition.

[36] Eriksson LS, Conn HO (1989). Branched-chain amino acids in the management of hepatic encepahalopathy: An analysis of variants. Hepatology.

[37] Wahren J, Denis J, Desurmont P, Eriksson LS, Escoffier JM, Gauthier AP (1983). Is intravenous administration of branched chain amino acids effective in the treatment of hepatic encephalopathy? A multicenter study. Hepatology.

[38] Kuhl DA, Brown RO, Vehe KL, Boucher BA, Luther RW, Kudsk KA (1990). Use of selected visceral protein measurements in comparison of branched-chain amino acids with standard amino acids in parenteral nutrition in injured patients. Surgery.

[39] von Meyenfeldt MF, Soeters PB, Vente JP, van Berlo CL, Rouflart MM, de Jong KP (1990). Effect of branched chain amino acid enrichment of total parenteral nutrition of nitrogen sparing and clinical outcome of sepsis and trauma: A prospective randomized double blind trial. Br J Surg.

[40] Lacey JM, Wilmore DW (1990). Is glutamine a conditionally essential amino acid?. Nutr Rev.

[41] Souba WW, Smith RJ, Wilmore DW (1985). Glutamine metabolism by the intestinal tract. JPEN J Parenter Enteral Nutr.

[42] Hammarqvist F, Wernerman J, Ali R, von der Decken A, Vinnars E (1989). Addition of glutamine to total parenteral nutrition after elective abdominal surgery spares free glutamine in muscle, counteracts the fall in muscle protein synthesis, and improves nitrogen balance. Ann Surg.

[43] Morlion BJ, Stehle P, Wachtler P, Siedhoff HP, Köller M, König W (1998). Total parenteral nutrition with glutamine dipeptide after major abdominal surgery: A randomized, double-blind, controlled study. Ann Surg.

[44] Jian ZM, Cao JD, Zhu XG, Zhao WX, Yu JC, Ma EL (1999). The impact of alanyl-glutamine on clinical safety, nitrogen balance, intestinal permeability, and clinical outcome in postoperative patients: A randomized, double-blind, controlled study of 120 patients. JPEN J Parenter Enteral Nutr.

[45] Goeters C, Wenn A, Mertes N, Wempe C, Van Aken H, Stehle P (2002). Parenteral L-alanyl-L-glutamine improves 6-month outcome in critically ill patients. Crit Care Med.

[46] McMahon MM (1995). Glucose vs.lipid as a calorie source. Proceedings of the ASPEN 19^th^ Clinical Congress, Miami, Jan 15-18, 1995.

[47] Wolfe RR, O'Donnell TF, Stone MD, Richmand DA, Burke JF (1980). Investigation of factors determining the optimal glucose infusion rate in total Parenteral nutrition. Metabolism.

[48] Michael SR, Sabo CE (1989). Management of the diabetic patient receiving nutritional support. Nutr Clin Pract.

[49] Fryburg DA, Gelfand RA (1990). Is exogenous fructose metabolism truly insulin independent?. JPEN J Parenter Enteral Nutr.

[50] Georgieff M, Moldawer LL, Bistrian BR, Blackburn GL (1985). Xylitol, an energy source for intravenous nutrition after trauma. JPEN J Parenter Enteral Nutr.

[51] Karlstad MD, DeMichele SJ, Bistrian BR, Blackburn GL (1991). Effect of total parenteral nutrition with xylitol on protein and energy metabolism in thermally injured rats. JPEN J Parenter Enteral Nutr.

[52] Seidner DL, Mascioli EA, Istfan NW, Porter KA, Selleck K, Blackburn GL (1989). Effects of long-chain triglyceride emulsions on reticulothelial system function in humans. JPEN J Parenter Enteral Nutr.

[53] Jensen GL, Mascioli EA, Seidner DL, Istfan NW, Domnitch AM, Selleck K (1990). Parenteral infusion of long- and medium-chain triglycerides and reticulothelial system function in man. JPEN J Parenter Enteral Nutr.

[54] Hyltander A, Sandström R, Lundholm K (1995). Metabolic effects of structured triglycerides in humans. Nutr Clin Pract.

[55] Baldermann H, Wicklmayr M, Rett K, Banholzer P, Dietze G, Mehnert H (1991). Changes of hepatic morphology during parenteral nutrition with lipid emulsions containing LCT or MCT/LCT quantified by ultrasound. JPEN J Parenter Enteral Nutr.

[56] Morlion BJ, Torwesten E, Lessire H, Sturm G, Peskar BM, Fürst P (1996). The effect of parenteral fish oil on leukocyte membrane fatty acid composition and leukotriene-synthesizing capacity in patients with postoperative trauma. Metabolism.

[57] Tappenden KA, Thomson AB, Wild GE, McBurney MI (1997). Shortchain fatty acid-supplemented total parenteral nutrition enhances functional adaptation to intestinal resection in rats. Gastroenterology.

[58] McCarthy MC, Turner WW, Whatley K, Cottam GL (1983). Topical corn oil in the management of essential fatty acid deficiency. Crit Care Med.

[59] Sacks GS, Brown RO, Collier P, Kudsk KA (1994). Failure of topical vegetable oils to prevent essential fatty acid deficiency in a critically ill patient receiving long-term parenteral nutrition. JPEN J Parenter Enteral Nutr.

[60] Brown DH, Simkover RA (1987). Maximum hang times for i.v fat emulsions. Am J Hosp Pharm.

[61] Henry RS, Jurgens RW, Sturgeon R, Athanikar N, Welco A, Van Leuven M (1980). Compatibility of calcium chloride and calcium gluconate with sodium phosphate in a mixed TPN solution. Am J Hosp Pharm.

[62] Hambidge KM, Sokol RJ, Fidanza SJ, Goodall MA (1898). Plasma manganese concentrations in infants and children receiving parenteral nutrition. JPEN J Parenter Enteral Nutr.

[63] Mehta R, Reilly JJ (1990). Manganese levels in jaundiced long-term total parenteral nutrition patient: potentiation of haloperidol toxicity?. JPEN J Parenter Enteral Nutr.

[64] Ejima A, Imamura T, Nakamura S, Saito H, Matsumoto K, Momono S (1992). Manganese intoxication during total parenteral nutrition. Lancet.

[65] Taylor S, Manara AR (1994). Manganese toxicity in a patient with cholestasis receiving total parenteral nutrition. Anaesthesia.

[66] Fredstrom S, Rogosheske J, Gupta P, Burns LJ (1995). Extrapyramidal symptoms in a BMT recipient with hyperintense basal ganglia and elevated manganese levels. Bone Marrow Transplant.

[67] Albina JE, Melnik G, Rombeau JL, Caldwell MD (1993). Fluid, electrolytes and body composition. Clinical nutrition: Parenteral nutrition.

[68] Kwong KW, Tsallas G (1990). Dilute iron dextran formulation for addition to parenteral nutrient solutions. Am J Hosp Pharm.

[69] Tu YH, Knox NL, Biringer JM, Eichman ML, Schweinsberg PD, Howard JR (1992). Compatibility of iron dextran with total nutrient admixture. Am J Hosp Pharm.

[70] Galley HF, Davies MJ, Webster NR (1996). Ascorbyl radical formation in patients with sepsis: effect of ascorbate loading. Free Radic Biol Med.

[71] Singer P, Berger MM, Van den Berghe G, Biolo G, Calder P, Forbes A (2009). ESPEN Guidelines on Parenteral nutrition: Intensive care. Clin Nutr.

[72] McMahon M, Manji N, Driscoll DF, Bistrian BR (1989). Parenteral nutrition in patients with diabetes mellitus: Theoretical and practical considerations. JPEN J Parenter Enteral Nutr.

[73] Finney SJ, Zekveld C, Elia A, Evans TW (2003). Glucose control and mortality in critically ill patients. J Am Med Assoc.

[74] Van den Berghe G, Wouters PJ, Bouillon R, Weekers F, Verwaest C, Schetz M (2003). Outcome benefit of intensive insulin therapy in the critically ill: Insulin dose versus glycemic control. Crit Care Med.

[75] Van den Berghe G, Wouters PJ, Bouillon R, Weekers F, Verwaest C, Schetz M (2001). Intensive insulin therapy in the critically ill patients. N Engl J Med.

[76] Cynober L, Prugnaud O, Lioret N, Duchemin C, Saizy R, Giboudeau J (1991). Serum transthyretin levels in patients with burn injury. Surgery.

[77] Chertow GM, Ackert K, Lew NL, Lazarus JM, Lowrie EG (2000). Prealbumin is as important as albumin in the nutritional assessment of hemodialysis patients. Kidney Int.

[78] Duggan A, Huffman FG (1998). Validation of serum transthyretin (prealbumin) as a nutritional parameter in hemodialysis patient. J Ren Nutr.

[79] Staun M, Pironi L, Bozzetti F, Baxter J, Forbes A, Joly F (2009). ESPEN Guidelines on Parenteral Nutrition: Home Parenteral Nutrition (HPN) in adult patients. Clin Nutr.

[80] Gendrel D, Bohuon C (2000). Procalcitonin as a marker of bacterial infection. Pediatr Infect Dis J.

[81] Solomon SM, Kirby DF (1990). The refeeding syndrome: A review. JPEN J Parenter Enteral Nutr.

[82] Brooks MJ, Melnik G (1995). The refeeding syndrome: an approach to understanding its complications and preventing its occurrence. Pharmacotherapy.

[83] Stegink LD, Freeman JB, Wispe J, Connor WE (1977). Absence of the biochemical symptoms of essential fatty acid deficiency in surgical patients undergoing protein sparing therapy. Am J Clin Nutr.

[84] Martin GR, Wallace LE, Sigalet DL (2004). Glucagon-like peptide-2 induces intestinal adaptation in parenterally fed rats with short bowel syndrome. Am J Physiol Gastrointest Liver Physiol.

[85] Jordan A, Stein J (2003). Effect of omega-3 fatty acid containing lipid emulsion alone and in combination with 5-fluorouracil (5-FU) on growth of the colon cancer cell line Caco-2. Eur J Nutr.

